# Maximum‐likelihood approaches reveal signatures of positive selection in BMP15 and GDF9 genes modulating ovarian function in mammalian female fertility

**DOI:** 10.1002/ece3.3336

**Published:** 2017-09-21

**Authors:** Hafiz Ishfaq Ahmad, Guiqiong Liu, Xunping Jiang, Shishay Girmay Edallew, Teketay Wassie, Birhanu Tesema, Yu Yun, Liu Pan, Chenhui Liu, Yuqing Chong, Zhao Jia Yu, Han Jilong

**Affiliations:** ^1^ Key Laboratory of Agricultural Animal Genetics Breeding and Reproduction of the Ministry of Education College of Animal Science and Technology Huazhong Agricultural University Wuhan China

**Keywords:** BMP15, evolution, GDF9, mammals, maximum likelihood, positive selection

## Abstract

Bone morphogenetic proteins (BMPs) and the growth factors (GDFs) play an important role in ovarian folliculogenesis and essential regulator of processes of numerous granulosa cells. BMP15 gene variations linked to various ovarian phenotypic consequences subject to the species, from infertility to improved prolificacy in sheep, primary ovarian insufficiency in women or associated with minor subfertility in mouse. To study the evolving role of BMP15 and GDF9, a phylogenetic analysis was performed. To find out the candidate gene associated with prolificacy in mammals, the nucleotide sequence of BMP15 and GDF9 genes was recognized under positive selection in various mammalian species. Maximum‐likelihood approaches used on BMP15 and GDF9 genes exhibited a robust divergence and a prompted evolution as compared to other TGFβ family members. Furthermore, among 32 mammalian species, we identified positive selection signals in the hominidae clade resulting to 132D, 147E, 163Y, 191W, and 236P codon sites of BMP15 and 162F, 188K, 206R, 240A, 244L, 246H, 248S, 251D, 253L, 254F and other codon sites of GDF9. The positively selected amino acid sites such as Alanine, Lucien, Arginine, and lysine are important for signaling. In conclusion, this study evidences that GDF9 and BMP15 genes have rapid evolution than other TGFß family members and was subjected to positive selection in the mammalian clade. Selected sites under the positive selection are of remarkable significance for the particular functioning of the protein and consequently for female fertility.

## 1 INTRODUCTION

Ovarian folliculogenesis is essential for mammalian female fecundity and is regulated by a composite endocrine network between the pituitary and the ovary. In this milieu, the intra‐ovarian paracrine communications are important in oocyte development and follicle evolution and maturation and provide the tolerable sensitivity to gonadotrophic inducement. The molecular machinery is necessary during oogenesis in oocyte preparation to support embryonic development (Sánchez & Smitz, 2012). However, the oocyte development within the follicular structure involves uninterrupted two‐way dialogs between the oocyte and cumulus complex, as well as the other somatic cells in the follicles, such as the granulosa and theca cells (Wigglesworth et al., 2013). The granulosa cells are important components of the follicular environment for the achievement of oocyte capability, ovulation, and fertilization as they regulate the expression of luteinizing hormone receptor (LHR), production of estradiol and progesterone, Inhibin A and B secretion, and production of several transcripts vital proteins (Ceko et al., 2014; Hatzirodos et al., 2014). The bone morphogenetic protein 15 and growth differentiation factor 9, belong to the TGF‐β superfamily, act on the granulosa cells to regulate oocyte growth and differentiation. These are expressed in all phases of follicle development in the mammalian species and are involved in steroidogenic regulation of granulosa cells (Dias, Khan, Adams, Sirard, & Singh, 2014; Peng et al., 2013). A recent phylogenetic analysis revealed that the GDF9 and BMP15 genes diverged promptly and showed fast evolution as compared to other BMPs. However, only BMP15 was acquiesced to a positive selection in the mammalian clade (Auclair et al., 2013). The candidate gene associated with prolificacy in goats, the most part nucleotide sequence of genes, including GDF9 and BMP15, were recognized in various goat breeds for their possible association to the high fertility (He, Ma, Liu, Zhang, & Li, 2010). As BMP15 and GDF9 play an important role in fertility and prolificacy, it therefore means that consideration should be made to the gene sequences (using both wet and dry lab methods), that are generally accountable for the detected phenotypic variations. A good understanding of these genes sequences will help in recognizing the modifications accountable for various factors ascribed to the gene. The objective of this study was to explore the selection signatures using maximum‐likelihood approaches on the bases of molecular genetic difference of BMP15 and GDF9 among mammalian species with a view to provide applicable genetic information for marker assisted selection in the different species.

## 2 MATERIAL AND METHODS

### 2.1 Sequence analysis and data set preparation

The coding nucleotide and amino acid sequences of BMP15 and GDF9 genes used in this analyses were recovered from GenBank (www.ncbi.nlm.nih.gov/genbank), Ensembl (http://useast.ensembl.org/index.html), and UniProt (http://www.uniprot.org), and recovered sequences were aligned using ClustalOmega, executed in MEGA 6.0 program (Tamura, Stecher, Peterson, Filipski, & Kumar, 2013), followed by manual adjustment. The phylogenetic tree of BMP15 and GDF9 genes was generated with MEGA 6.0 based on maximum‐likelihood method. The taxa clustered together in the bootstrap test 1,000 replicates based on maximum‐likelihood method selecting the topology with higher log likelihood value and the branch length measured in the number of substitutions per site. (Ahmad et al., 2017; Asif, Awais, Qadri, Ahmad, & Du, 2017). The accession numbers and identification of species used for BMP15 and GDF9 are listed in Table S1.

### 2.2 Codon‐based positive selection analysis

In order to recognize particular codons under positive selection of mammalian BMP15 and GDF9 sequences, the different ω ratios (dN/dS) were compared using two maximum‐likelihood approaches, the HyPhy package implemented in the DATAMONKEY Web Server (http://www.datamonkey.org/) (Poon, Frost, & Pond, 2009) and CODEML implemented in PAML version 4 (Yang, 2007) being considered in the analysis the results where ω ratios were significantly higher than 1.

The analysis involves of two main steps. In the first step, we used the maximum‐likelihood ratio test to find out positive selection, that is, manifestation of sites with ω > 1. We achieved this by comparing a (null) model that does not allow for sites with ω > 1 and a general (discrete) model that does. The likelihood log (2Δl) is compared with *df* = 4 with the χ^2^ distribution. The M7 model (null) postulates a β distribution with ω restricted in the interval (0 and 1). The M8 model (ω and beta) is an alternative model includes two parameters, so ω value obtained from the data set can be greater than one. Positive selection clues in BMP15 and GDF9 genes were identified by calculating the rates of synonymous and nonsynonymous variations at each site in alligned sequence using different likelihood tests such as fixed effect likelihood (FEL), random effect likelihood (REL), and single likelihood ancestor counting (SLAC) methods (Ahmad et al., 2017) by estimating global ω values.

The second main step is to find out amino acid subjected to positive selection when their presence is confirmed by likelihood test. It is inferred by using the Bayes theorem to estimate the posterior probabilities for each site, from the different ω classes (Bielawski & Yang, 2003). The amino acid residues with high probabilities having ω > 1 are probably found to be under selection. Amino acid locations subjected to positive selection were drawn onto the crystal structure using Phyre (http://www.sbg.bio.ic.ac.uk/phyre2/html) and Swiss model (http://swissmodel.expasy.org) online programs (Kelley & Sternberg, 2009). The level of evolutionary conservation amino acid/nucleic acid positions in protein was predicted using the bioinformatics tool, the ConSurf server (http://consurftest.tau.ac.il) based on phylogenetic relationship between sequences (Glaser et al., 2003). To further ratify codon sites under the selection pressure, aligned codon sequence of BMP15 and GDF9 was tested in the Selecton, version 2.2 (http://selecton.tau.ac.il/) that allows shifting the ω ratio between different codons within the aligned sequence and this was measured by maximum‐likelihood test through Bayesian inference method (Yang, Liao, Zhuang, & Zhang, 2012). Moreover, the selecton results are shown with color scales demonstrating various types of selection.

### 2.3 Protein–protein interaction network analysis

To further expose the molecular functioning mechanisms of BMP15 and GDF9, we recognized the vital genes interacted with BMP15 and GDF9 followed by protein–protein interaction linkage analysis sing STRING (version 9.1, http://www.string-db.org/) (Franceschini et al., 2012) which is web server and biological databank which comprises widely anticipated and identified interaction data. The interactions between protein encoded by the BMP15 and GDF9 were sought. The pooled score <0.4 was used as the cutoff standard. The bioinformatics databank as an open access source comprises interactions of proteins involved in various pathways. The middle nodes indicate the protein which own essential biological function and are highly connected, were identified by estimating the betweenness value and the number of line connections between proteins of each node. The network was constructed using STRING and was visualized by Cytoscape software (http://www.cytoscape.org/) (Li, Zhao, Wang, Zong, & Yang, 2017).

## 3 RESULTS

The average ω ratio (dN/dS) across the sites and lineage are <1 for BMP15 and GDF9 (Table 1). However, these proteins subjected to positive selection and might have conserved amino acid exposed to purifying selection and have ω less than one. The level of evolutionary conservation amino acid/nucleic acid positions in protein was predicted using the ConSurf server (http://consurftest.tau.ac.il) based on phylogenetic relationship between sequences and Selecton version 2.2 that implements the mechanistic empirical combination (MEC) model for estimating adaptive selection pressure at different codons. A huge number of conserved amino acids would mask the positive selection signals, and we found positive selection on variable amino acids which were exposed or buried residues according to the neural network algorithm BMP15 and GDF9.

**Table 1:** Log likelihood values and test statistics for PAML site models of positive selection

Gene	No. of residues	No. of species	Model	Parameter estimates	*lnL* M7	*lnL* M8	*2ΔlnL*	PAML	FEL	REL	SLAC	% of sites
BMP15	1234	37	M7	*p* = .60414, *q* = 0.76298	−19,737.9	−19,329.1	17.69**	**40**,** 49**, 75, 90, **109**,** 133**, 176, 191, 199,	17, 126, 144, 148,	45, 95, 126, 144,	45, 384, 386	0.53
			M8	P0 = 0.94697, *p* = .66983, *q* = 0.97138				200, 203, 258, 289,	248, 363	363, 384		
				P1 = 0.05303, **ω1 = 1.56019**				297, 315, 323, 365				
GDF9	1371	32	M7	*p* = .48463, *q* = 0.91798	−13,717.5	−13,716.2	2.59*	30, 186, **245**, 254,	21, 30, 34, 139, 186	21, 30, 186, 299,	21, 30, 186, 299,	0.78
			M8	P0 = 0. 99219, *p* = .49979, *q* = 0. 97580				**292**, 302, *304*	299, 304, 339	304, 339	304	
				P1 = 0.00781, **ω1 = 1.93407**								

The proportion of sites under positive selection (p1), or under selective constraint (p0), and parameters *p* and *q* for the beta distribution. Parameters indicating positive selection are in bold. *p*: significant at 5% level; *p*: significant at 1% level. Sites potentially under positive selection identified under model M8 are listed according to the human sequence numbering. Positively selected sites with posterior probability 0.9 are italicized, 0.8–0.9 in bold, and 0.5–0.7 in plain text. The test statistic 2Δl is compared to a χ^2^ distribution with 2 *df*, critical values 5.99, 9.21, and 13.82 at 5%, 1%, and 0.1% significance, respectively.

**Significant at 1% level; *Significant at 5% level.

As a refined selection test, M8 was compared with M7. M8 was significant and fit the data more significantly than M7. We found positive selection for BMP15 and GDF9 proteins under model M8. The proportion of sites under M8 are 0.53% with ω = 1.56 for BMP15 and 0.78% with ω = 1.93 for GDF9. The proportion of positively selected sites under M8 is clear statistical evidence positive Darwinian selection (Table 1). There were 17 codon sites of BMP15 and 7 sites of GDF9 under positive selection in various likelihood approaches. Evolutionary signal of positive selection was inferred by computing global ω values using FEL, REL, and SLAC tests. The results revealed that there was robust sign of positive selection in BMP15 and GDF9 genes in mammals. The FEL and REL detected six sites and SLAC identified three sites under the positive selection for BMP15 at various codon positions, respectively (Table 1), while FEL, REL, and SLAC analyses detected eight, six, and five sites under positive selection, respectively (Table 1). REL detected positive sites at 95% confidence interval. REL analysis detected sites under positive selection which used Bayes factor, and the values > 20. *p* Values < .05 were measured as significant for other analysis and all identified sites were considerably different and having *p*‐values < .05.

### 3.1 Positive selection on amino acid positions

**Figure 1:**
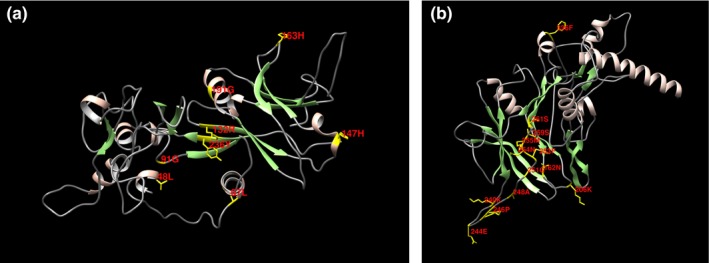
(a) Location of positively selected amino acid sites identified BMP15 gene. The crystal structure of human BMP as a reference and positively selected sites were drawn onto the crystal structure using Phyre tool (http://www.sbg.bio.ic.ac.uk/phyre2/html). All the residues identified as under selection fall in the domain containing the ligand binding site. The sites which fall in the region identified as the ligand binding site and another cluster in a region immediately following the signal sequence. (b) Location of positively selected amino acid sites identified GDF9 gene. The crystal structure of human GDF9 as a reference and positively selected sites was drawn onto the crystal structure using Phyre tool (http://www.sbg.bio.ic.ac.uk/phyre2/html). All the residues identified as under selection fall in the domain containing the ligand binding site. The sites which fall in the region identified as the ligand binding site and another cluster in a region immediately following the signal sequence

**Figure 2:**
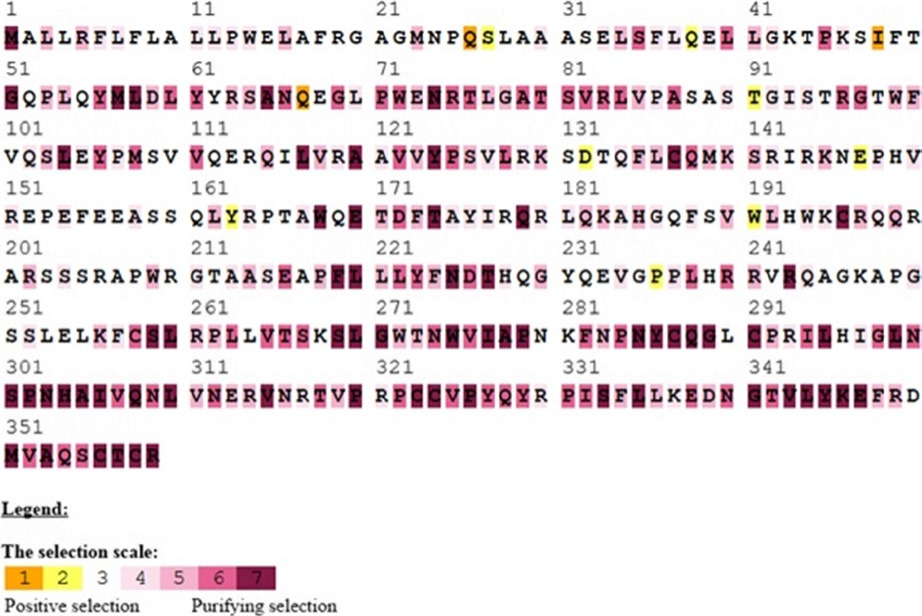
Selection pressures among goat BMP15 gene sequences using mechanistic empirical combination (MEC) model of selecton online tool. Yellow and brown highlights represent positive selection, gray and white highlights represent neutral selection, and purple highlight represents negative selection on codons

**Figure 3:**
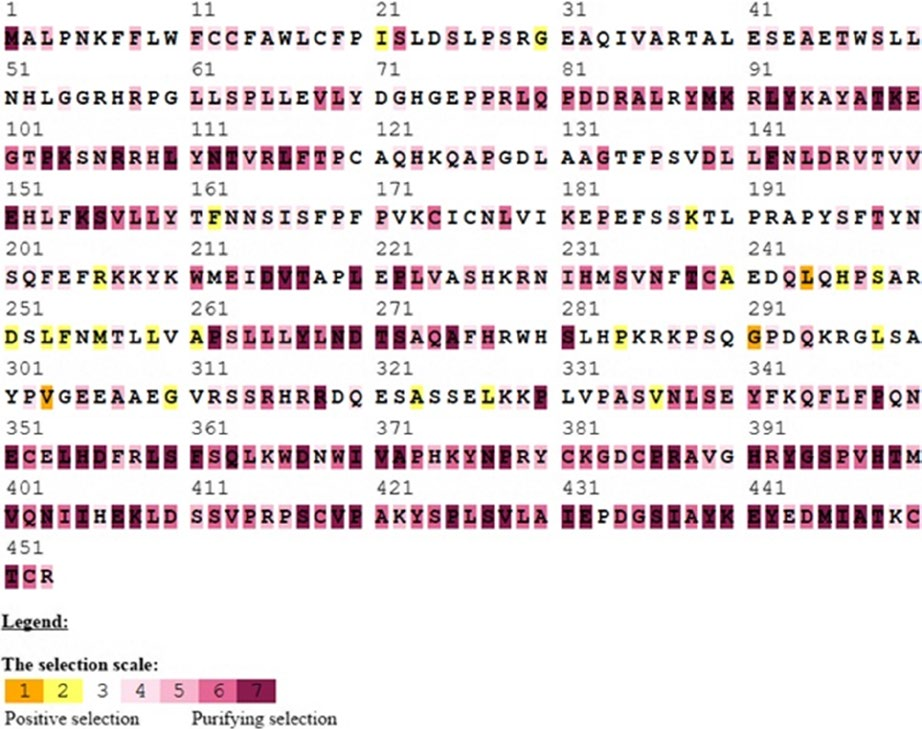
Selection pressures among goat GDF9 gene sequences using mechanistic empirical combination (MEC) model of selecton online tool. Yellow and brown highlights represent positive selection, gray and white highlights represent neutral selection, and purple highlight represents negative selection on codons

Indicating the positions of amino acids evolutionary conservation is important for maintaining the protein structure and function. Therefore, detection of selected sites may enlighten the selection forces and detects the functionally significant sites for bone morphogenetic protein interaction. To detect such sites, we utilized the Bayes method to estimate the posterior probabilities for each site. The sites with more probabilities are expected to be positively selected with ω > 1. Using Using BEB analysis for 391 amino acids of BMP15, seventeen were found under positive selection but no site could be identified at 99% or 95% posterior probability. GDF9 had 453 amino acid sites, and only seven amino acids showed positive selection (Table 2; Figure 1a,b). Regarding PAML false positive results, we also performed positive selection test in the selecton server (http://selecton.tau.ac.il/) that uses the Mechanistic Empirical Combination (MEC) model for estimating the selection pressure at particular codons. The MEC model takes into account the variances between amino acid substitution rates. Adaptive selection pressure was found at various codons in BMP15 (Figure 2) and GDF9 (Figure 3), identified under positive selection.

**Table 2:** Positively selected sites under different PAML site models using bayes empirical bayes analysis

Gene	Model	Codon	Amino acid	Posterior probability	Post mean ± *SE* for ω
BMP15	M8: selection, beta+ ω	40	V	0.846	1.407 ± 0.22
		49	I	0.678	1.303 ± 0.29
		75	Q	0.755	1.350 ± 0.27
		90	R	0.923	1.455 ± 0.16
		109	A	0.824	1.393 ± 0.24
		133	V	0.888	1.434 ± 0.19
		176	H	0.55	1.218 ± 0.32
		191	P	0.703	1.315 ± 0.29
		199	G	0.567	1.224 ± 0.32
		200	R	0.651	1.281 ± 0.31
		203	P	0.607	1.249 ± 0.32
		258	W	0.572	1.227 ± 0.32
		289	K	0.609	1.253 ± 0.32
		297	K	0.765	1.358 ± 0.26
		315	A	0.727	1.331 ± 0.28
		323	R	0.775	1.362 ± 0.26
		365	V	0.578	1.238 ± 0.31
GDF‐9	M8: selection, beta+ ω	30	G	0.774	1.576 ± 0.39
		186	F	0.581	1.270 ± 0.34
		245	L	0.814	1.185 ± 0.34
		254	L	0.722	1.242 ± 0.34
		292	G	0.824	1.203 ± 0.34
		302	Y	0.531	1.350 ± 0.36
		304	V	0.958^a^	1.271 ± 0.33

a Posterior probabilities >90%.

### 3.2 Protein–protein interaction network

**Figure 4:**
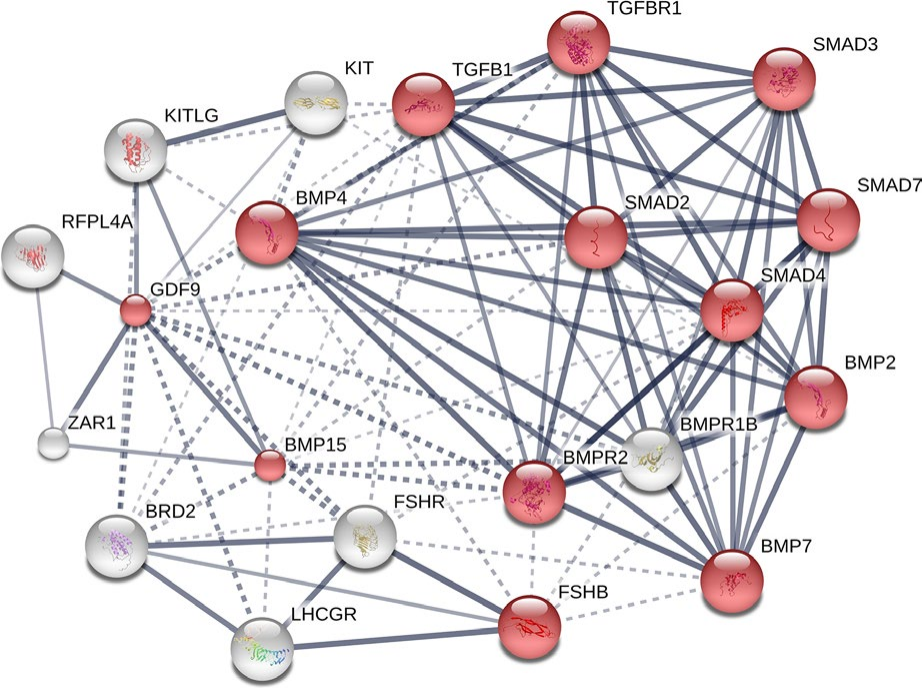
The protein–protein interaction (PPI) network built by STRING database for BMP15 and GDF genes. Gray and Red circles characterize downregulated and upregulated genes, respectively. Line thickness indicates the strength of the interaction. Dash and solid edge mean negative and positive correlation coefficient. Network nodes denote proteins' post‐transcriptional modifications or splice isoforms, and each node represents all the proteins produced by a single, protein‐coding gene locus

By searching BMP15 and GDF9 encoded protein to the STRING databank, various PPI pairs were found. The PPI network had 21 nodes (denote BMP15 and GDF9 encoded proteins) and 102 edges (line networks between nodes) (Figure 4). In the PPI network, BMP15 and GDF9 are interacted with the other reproductive key genes which are co‐expressed. We found 19 genes: KITLG, KIT, TGFβ, TGFβR, SMAD2, SMAD3, SMAD4, SMAD7, BMP2, BMP4, BMP7, FSHβ, LHCGR, ZAR1, BRD2, BMPR2, and BMPR1β (Figure 4). Among these, TGFβ, TGFβR, SMAD2, SMAD3, SMAD4, SMAD7, BMP2, BMP4, BMP7, and FSHβ are vital genes involved in biological signaling pathways in reproduction because these are upregulated genes with BMP15 and GDF9 (Figure 4). The molecular pathways of all the interacted proteins are involved in various reproductive functions such as TGF beta binding receptor, cytokine receptor binding, SMAD binding, and others proteins are interconnected with each other in various biological molecular pathways that are involved in modulating the reproductive efficiency in mammals.

## 4 DISCUSSION

The BMP15 and GDF9 contribute in the development of primary follicle from primordial follicle and play an essential role in the subsequent phases of follicular growth and maturation, enhancing the expression of luteinizing hormone receptor (LHR), steroidogenic regulatory protein, and plasminogen activators. These factors are also involved in the communication among oocyte and cumulus cells, where they control the biosynthesis of cholesterol in cumulus cells, glycolysis, and absorption of amino acids. Although the mode of action has not been fully understood, in vitro studies revealed that the factors BMP15 and GDF9 stimulate the ovarian follicles growth and cumulus cells proliferation through the initiation of mitosis in cells and theca and granulosa expression of genes connected to follicular maturation (de Castro, Cruz, & Leal, 2016). Assumed that positive selection is generally linked with the branch length and rapid gene evolution and the genes related to reproduction evolve rapidly and often proved positive selection (Meslin et al., 2012). We accomplished positive selection analyses on BMP15 and GDF9. We used coding nucleotide sequences of 32 mammalian species which were evaluated by branch‐site models in PAML package (Yang, 2007), in order to investigate whether the diverse species in the phylogenetic kinship experienced selection pressure and to identify clues of native periodic positive selection. These evaluates were performed on the complete sequences, the mature, and the pro‐region form of BMP15 and GDF9. We studied all branches of the phylogenetic tree, and various codon sites were found under positive selection in mammalian clade (Figure 1a,b and Table 1). We identified fifteen positively selected sites with PAML, six sites with FEL and REL, and four sites with SLAC models for BMP15; eleven of these fifteen amino acids (Figure 2) were also recognized by the estimation of positive selection using the mechanistic empirical combination (MEC) model for measuring adaptive selection pressure at codons on the BMP15 sequence. We identified seven positively selected sites with PAML, eight with FEL, six with REL, and four with SLAC model for GDF9. Furthermore 22 amino acids were found positively selected for the hominidae clade, corresponding to human coding sequences (Figure 3) using the mechanistic empirical combination (MEC) model in Selecton server. It has been testified that GC influenced gene conversion might rise the dN/dS ratio, particularly in primates and yield false‐positive recognition of amino acids in branch‐site model of positive selection (Ratnakumar et al., 2010). It has been revealed that in hominidae clade, the third codon position of BMP15, the G‐C content (53%) is higher than the gene in these taxa (46%) (Romiguier, Ranwez, Douzery, & Galtier, 2010). However, a current study (Gharib & Robinson‐Rechavi, 2013) describes that the G‐C rate of a sequence did not considerably interfere the branch‐site model. A phylogenetic analysis was performed to study the evolving role of BMP15. A maximum‐likelihood phylogenetic kinship of mammalian BMP15 expressed by the ovary exhibited that BMP15 has a very robust divergence and a fast evolution compared to other genes. Furthermore, positive selection signals detected in the *hominidae* clade consistent to F146, L189 and Y235 codon sites in human BMP15 (Auclair et al., 2013). We performed selection analyses to investigate amino acid positions under the positive selection through Bayes Empirical Bayes and found various amino acid sites of BMP15 and GDF9 under positive selection. We found positive selection signals at 131D, 163Y, 191W, 147E, and 236P codon sites of BMP15 (Figure 2) and 162F, 188K, 206R, 240A, 244L, 246H, 248S, 251D, 253L, 254F, and other codon sites of GDF9 (Figure 3). The positively selected amino acid sites such as alanine, leucine, arginine, and lysine are important for signaling. Among 24 mammalian species, positive selection signals were detected signals in the human BMP15 (Auclair et al., 2013). Therefore, some amino acid sites under positive selection are essential for particular role of protein and consequently for female fertility (Persani, Rossetti, Di Pasquale, Cacciatore, & Fabre, 2014). Moreover, the transformed was more effective than wild type in deterring the progesterone production in granulosa cells of ovine cell culture. It is evidenced that BMP15 has evolved faster than other TGF family members and was acquiesced to positive selection in mammalian clade (Persani et al., 2014). The sequence alignment reveals that BMP15 belongs to the TGF family of cytokines due to the existence of “cystine‐knot” motif, together with GDF9 as the next homolog. Like other TGF members, these molecules are first decoded as signal peptide with a pre‐pro‐peptide at N‐terminal followed by pro‐domain and the C‐terminal mature sphere that delivers the biological action (Chang, Brown, & Matzuk, 2002). The particular functions of TGF superfamily member's pro‐domains are unidentified. The proteolytically treated pro‐region and mature regions of BMPs remain attached noncovalently, usually networking with the extracellular matrix (Sengle, Ono, Sasaki, & Sakai, 2011). Regarding the BMP15 pro‐region, it drives the dimerization and subsequent secretion of the mature dimers and may help to alleviate the mature region bioactivity (Pulkki et al., 2011). In our study, positive selection at BMP15 and GDF9 was found with ω > 1 (Table 1). This indicates that nonsynonymous (dN) sites evolved quicker than those of synonymous sites and positive Darwinian selection influence purifying/balancing selection favored new variants and raised allelic polymorphism (Bergström & Gyllensten, 1995) which in turn might introduce an alteration in protein structure validation, thus affecting the signaling pathways (Cui et al., 2009). The changing amino acid substitutions across species might be the result of discrete divergence from their common lineages, which agrees with former submissions. The orthologs differ from their most recent common forebear have different evolutionary routes which may direct the deviations in the selective constraints on homologous sites (Marini, Thomas, & Rine, 2010). Our analysis of bone morphogenetic protein genes involved in recent selection provides insights of some biological processes that have been objectives of selection in current and much longer evolutionary timescales (Voight, Kudaravalli, Wen, & Pritchard, 2006). Hence, understanding the story of selection in mammalian genome promises to be an interesting research area for years to come.

## 5 CONCLUSIONS

In present study, we investigated that BMP15 and GDF9 genes have evolved rapidly than other TGFß superfamily members and was allowed to selection pressure in mammalian clade. Some positively selected amino acid sites are of significant for the particular role of protein and consequently for female fertility. We presented comprehensive analyses in determination of genetic importance of BMP15 and GDF9. Selection analyses of bone morphogenetic proteins modulating reproduction could facilitate the development of unique strategies that may help for genetic improvement and select individuals with high breeding values for traits of interest as parentages to produce the next generation.

## CONFLICT OF INTEREST

None declared.

## AUTHOR CONTRIBUTION

HIA wrote original draft of manuscript. SGE, TW, BT, YY, LP, CL, YC, ZJY, and HJ help in data analysis. GL and XJ supervised the study.
