# Supplementary material for: Maximum‐likelihood approaches reveal signatures of positive selection in BMP15 and GDF9 genes modulating ovarian function in mammalian female fertility
Source: Ecol Evol. 2017 Sep 21;7(21):8895–902. doi: 10.1002/ece3.3336 (PMC5689494; doi:10.1002/ece3.3336)
Supplement: Supplementary file 1 [file ECE3-7-8895-s001.docx]

Table S1: NCBI GenBank accession numbers for BMP15 and GDF9 data sets. List of species and NCBI GenBank accession numbers for sequences used to construct the datasets for hypothesis testing. Species and accession numbers for each dataset are grouped together on the table.

|  | BMP15 |  | GDF9 |
| --- | --- | --- | --- |
| Species | Accession Number | Species | Accession Numbers |
| *Homo sapiens* | NM_005448.2 | *Homo sapiens* | NM_005260.5 |
| *Rattus norvegicus* | NM_021670.1 | *Mus musculus* | NM_008110.2 |
| *Mus musculus* | NM_009757.4 | *Capra hircus* | NM_001285708.1 |
| *Ovis aries* | NM_001114767.1 | *Bos taurus* | NM_174681.2 |
| *Bos taurus* | NM_001031752.1 | *Sus scrofa* | NM_001001909.1 |
| *Sus scrofa* | NM_001005155.1 | *Rattus norvegicus* | NM_021672.1 |
| *Bubalus bubalis* | XM_006059485.1 | *Felis catus* | NM_001165900.1 |
| *pigtailed macaque* | XM_011761998.1 | *Macaca nemestrina* | XM_011716609.1 |
| *Papio anubis* | XM_003917722.2 | *Cercocebus atys* | XM_012090234.1 |
| *Chlorocebus sabaeus* | XM_007991731.1 | *Bubalus bubalis* | XM_006050555.1 |
| *Panthera tigris altaica* | XM_007090093.1 | *Ailuropoda melanoleuca* | FJ546460.1 |
| *Pan paniscus* | XM_003805950.2 | *Callithrix jacchus* | XM_002744603.3 |
| *Sorex araneus* | XM_003640274.3 | *Papio anubis* | NM_001168763.1 |
| *Canis lupus familiaris* | XM_003640274.3 | *Nannospalax galili* | XM_008849331.1 |
| *Echinops telfairi* | XM_004713136.2 | *Manis javanica* | XM_017652511.1 |
| *Capra hircus* | NM_001285588.1 | *Cebus capucinus* | XM_017532429.1 |
| *Sorex araneus* | XM_004606466.2 | *Monodelphis domestica* | XM_001371982.3 |
| *Condylura cristata* | XM_004690047.2 | *Erinaceus europaeus* | XM_007519919.2 |
| *Felis catus* | NM_001165898.1 | *Rousettus aegyptiacus* | XM_016118390.1 |
| *Cebus capucinus imitator* | XM_017514205.1 | *Peromyscus maniculatus* | XM_006995887.2 |
| *Lipotes vexillifer* | XM_007450928.1 | *Macaca fascicularis* | XM_005557754.2 |
| *Tarsius syrichta* | XM_008055934.1 | *Vicugna pacos* | XM_006212821.2 |
| *Callithrix jacchus* | XM_002762884.2 | *Oryctolagus_cuniculus* | NM_001171350.1 |
| *Pongo abelii* | XM_002831651.2 | *Equus asinus* | XM_014856804.1 |
| *Ailuropoda melanoleuca* | XM_002930506.2 | *Equus caballus* | XM_001504427.3 |
| *Monodelphis domestica* | XM_007507736.2 | *Camelus ferus* | XM_006179602.1 |
| *Miniopterus natalensis* | XM_016218522.1 | *Bos mutus* | XM_005890431.2 |
| *Pteropus alecto* | XM_006913404.1 | *Canis lupus* | NM_001168013.1 |
| *Myotis davidii* | XM_006754222.2 | *Cavia porcellus* | XM_003464506.3 |
| *Vicugna pacos* | XM_006217832.2 | *Mesocricetus auratus* | XM_013111773.1 |
| *Oryctolagus cuniculus* | NM_001199117.1 |  |  |
| *Acinonyx jubatus* | *XM_015074758.1* | |  |
| *Equus asinus* | *XM_014827162.1* | |  |
| *Mustela putorius furo* | *XM_004755127.2* | |  |
| *Propithecus coquereli* | *XM_012642808.1* | |  |
| *Camelus dromedarius* | *XM_010998065.1* | |  |
